# The complete chloroplast genome of *Rhododendron molle* and its phylogenetic position within Ericaceae

**DOI:** 10.1080/23802359.2021.1959458

**Published:** 2021-08-09

**Authors:** Yan Liu, Qiaoyun Li, Ling Wang, Linshi Wu, Yaqi Huang, Juan Zhang, Yin Song, Juyang Liao

**Affiliations:** Hunan Botanical Garden, Changsha, PR China

**Keywords:** *Rhododendron molle*, chloroplast genome, phylogenetic analysis, Ericaceae

## Abstract

*Rhododendron mole* (Blume) G. Don is an attractive ornamental and valuable medicinal plant which widely distributed in the southern regions of China. In order to promote the studies on the genetic diversity of this species, we assembled the complete chloroplast (cp) genome of *R*. *molle* by using the genome skimming approach. The results showed that the cp genome of *R*. *molle* exhibited a quadripartite cycle with 197,877 bp, comprising of two inverted repeats (IRs) of 43,831 bp separated by a large single copy (LSC) region of 110,189 bp and a quite small single copy (SSC) region of 26 bp. It encodes 146 genes, including 92 protein-coding, 46 tRNA, and eight rRNA genes. The overall GC content of the cp genome was 36.0%. The phylogenetic analysis indicated that *R*. *molle* is closely related to *R*. *delavayi*. Thus, the cp genome sequence of *R*. *molle* provides a rich source of genetic information for studies on *Rhododendron* taxonomy, phylogeny, and evolution, as well as lays the foundation for further development and utilization of *R*. *molle*.

*Rhododendron molle* (Blume) G. Don (Ericaceae) is a deciduous shrub, which is mainly distributed in the southern regions of China (Wu et al. 2005). With flourishing branches and gorgeous flowers, it is one of the elite parents for new varieties improvement in *Rhododendron*. Moreover, previous studies suggested that different organs and tissues of *R*. *molle* (including flower and root, etc.) could be used as analgesics and insecticides (Zhong et al. 2006; Li et al. 2020; Zhang et al. 2020). Due to lacking genome information and available molecular markers, the phylogenetic relationship of *R*. *molle* with other related species is still unclear. Herein, we assembled the cp genome of *R*. *molle* and inferred its phylogenetic position in family Ericaceae which will be conducive to promoting the studies on the phylogeography, genetic diversity, and utilization of this species.

Fresh leaves of *R*. *molle* were collected from Changsha, Hunan, China (28°6′3′′N 113°12′22′′E, 75 m). The voucher specimen (CSFI036611) was deposited at Central South University of Forestry and Technology (https://www.cvh.ac.cn/spms/detail.php?id=f0cda901, Lei Wu, wuleiibk@163.com; 346204740@qq.com). The genomic DNA was isolated using the DNeasy Plant Mini Kit (QIAGEN GmbH, Hilden, Germany). A paired-end library of 2 × 150 bp and insert size of ∼350 bp was constructed according to the Illumina standard library, which was then sequenced on the Illumina HiSeq 2500 platform at Genepioneer Biotechnologies Inc. (Nanjing, China). About 4.9 GB clean data were used to assemble the cp genome using SPAdes (Bankevich et al. 2012). The cp genome of *R*. *delavayi* (MN711645.3) was used as a reference for further adjustments and then annotated using Dual Organellar Genome Annotator (DOGMA) (Wyman et al. 2004). The sequence of cp genome was deposited in GenBank (accession number: MZ073672).

The cp genome of *R*. *molle* was 197,877 bp in length, consisting of a pair of inverted repeats (IRs), a large single copy (LSC), and a small single copy (SSC), and the sequence lengths were 43,831 bp, 110,189 bp, and 26 bp, respectively. The overall GC content of the cp genome was 36.0%, while the GC percentages in IR, LSC, and SSC were 36.9, 35.3, and 7.7%, respectively. The genome encodes 146 genes, including 92 protein-coding, 46 tRNA, and eight rRNA genes.

To investigate the phylogenetic position of *R*. *molle*, the cp genome of 11 species belong to Ericaceae and Actinidiaceae (outgroup) were obtained. Coding sequences shared by all species were multiple aligned with MAFFT (Katoh and Standley 2013) and connected. The maximum-likelihood (ML) tree was reconstructed using RAxML (Stamatakis 2014) with the GTRGAMMA model and 1000 bootstrap replicates. The result showed that *R*. *molle* was closely related to *R*. *delavayi* (Figure 1).

**Figure 1.**
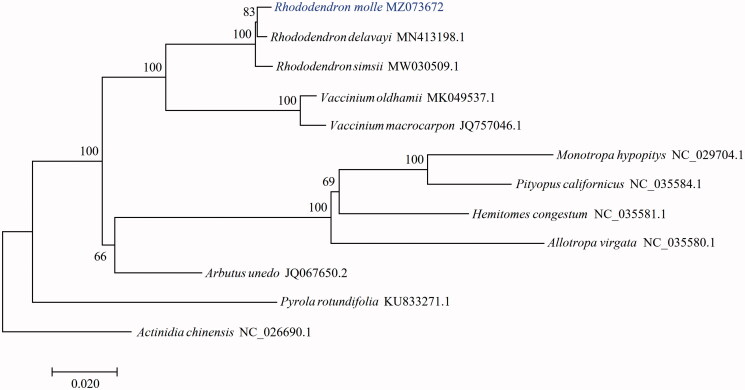
Maximum-likelihood phylogenetic tree for *Rhododendron molle* based on coding sequences shared by all species. *Actinidia chinensis* (NC_026690.1) were used as outgroup. The support values are shown at the branches.

## Data Availability

The data that support the findings of this study are openly available in GenBank, National Center for Biotechnology Information (NCBI) at https://www.ncbi.nlm.nih.gov/genbank/, with accession number of MZ073672. The associated BioProject, SRA, and Bio-Sample numbers are PRJNA742763, SRR15010628, and SAMN19967795, respectively.
